# Place of High-Flow Oxygen Therapy (HFOT) in the Perioperative Period: A Thoracic Surgery Case Report

**DOI:** 10.7759/cureus.89839

**Published:** 2025-08-11

**Authors:** Walid Atmani, Ayoub Boubkri, Hamza Najout, Hicham Balkhi, Mustapha Bensghir

**Affiliations:** 1 Department of Anesthesia and ICU, Mohammed V Military Teaching Hospital, Mohammed V University, Rabat, MAR

**Keywords:** high-flow oxygen therapy, preoxygenation, thoracic anesthesiology, thoracic surgery, ventilation apnea

## Abstract

High-flow oxygen therapy (HFOT) has emerged as a promising approach in perioperative respiratory management, particularly in preventing postoperative respiratory complications, although its application in thoracic surgery remains underexplored. We report the case of a 58-year-old female with a history of pulmonary tumor and metastatic bone disease who was admitted for thoracic surgery due to dyspnea (NYHA class III). Preoperative evaluation revealed poor general condition with stable hemodynamics. Given the elevated risk of respiratory complications, HFOT was initiated for both preoxygenation and apneic oxygenation during intubation. The patient underwent thoracoscopy with talc pleurodesis and was managed with intraoperative mechanical ventilation, followed by postoperative HFOT to prevent respiratory distress and support the transition to spontaneous breathing. HFOT effectively maintained oxygen saturation (SpO₂ 98-100%) throughout the perioperative period, with no episodes of hypoxemia or respiratory complications post-extubation. The patient remained stable, avoided reintubation, and was transferred to the thoracic surgery unit without requiring intensive care. This case suggests that HFOT may enhance oxygenation, reduce the risk of postoperative respiratory failure, and facilitate smoother recovery in thoracic surgery. Further studies are warranted to better define its role in this context.

## Introduction

The reduction of perioperative and postoperative mortality and morbidity is the primary goal of the anesthesiologist-intensivist in the management of patients in perioperative medicine. According to the latest available data, more than 10% of surgical patients will experience at least one postoperative pulmonary complication [1]. The risk of pulmonary complications increases with the presence of comorbidities, as well as with longer and more invasive surgeries [2]. Several complementary tools are available to physicians, such as protective ventilation during the intraoperative period [3] or non-invasive ventilation (NIV) [4], to prevent and promptly treat atelectasis. High-flow nasal oxygen therapy (HFOT) is a relatively recent technique that is increasingly integrated into the overall management of surgical patients [5]. HFOT has gained growing interest in recent years. Its clinical applications have multiplied, and numerous studies have investigated its potential benefits in various situations: in critical care for hypoxemic patients, during post-extubation, postoperatively, and even in the operating room [6]. The COVID-19 pandemic has also accelerated the adoption of this technique [7]. Thoracic surgery poses a significant challenge in anesthesia and postoperative care due to the heightened risk of respiratory complications, including hypoxemia, atelectasis, and acute respiratory failure. These complications are particularly common in patients with underlying respiratory conditions, such as chronic obstructive pulmonary disease (COPD), or in those requiring extensive pulmonary resections. In this context, HFOT has emerged as an innovative and effective therapeutic option. In this article, we present a concrete example of HFOT use in thoracic surgery patients, spanning preoxygenation, apneic ventilation, and postoperative care during extubation, thereby avoiding reintubation and facilitating the transition to spontaneous ventilation.

## Case presentation

**Figure 1:**
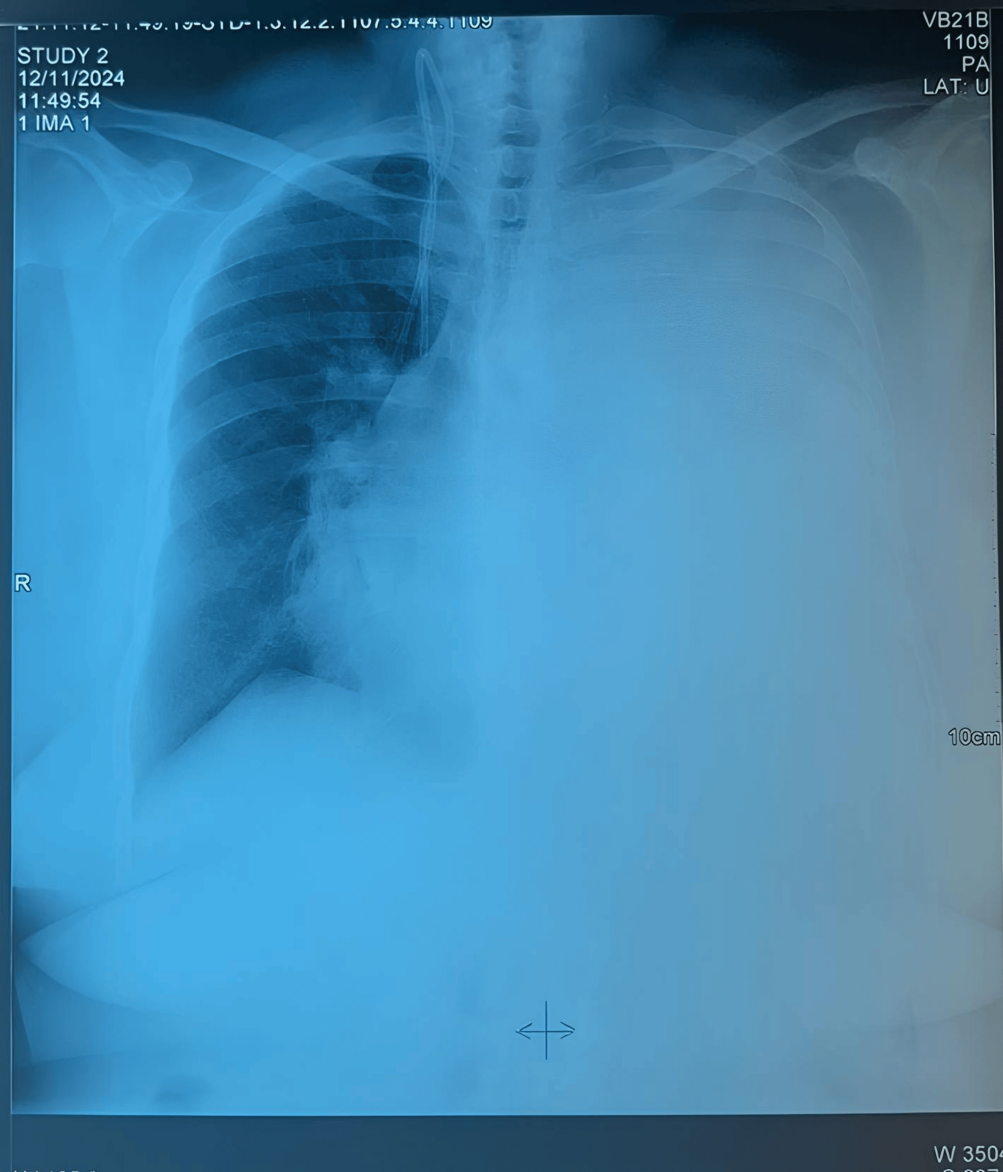
Left lung opacity due to large pleural effusion

A 58-year-old female patient with a history of a pulmonary tumor with bone metastases and high blood pressure managed by amlodipine was admitted to the thoracic surgery department for NYHA class III dyspnea. Clinical examination revealed a patient in poor general condition, afebrile, and conscious. Hemodynamically, her blood pressure (BP) was 110/65 mmHg, and her heart rate (HR) was 120 beats per minute. The respiratory examination showed a tachypneic patient with shallow breathing. Peripheral oxygen saturation (SpO₂) in ambient air was 90%. An initial standard chest X-ray (Figure 1) revealed a white area in the left lung.

A pleural tap was performed, providing relief with the evacuation of two liters of pleural fluid. The patient became eupneic under 3 L/min of oxygen via nasal cannula, with an SpO₂ of 98%. Talc pleurodesis was indicated, requiring an anesthetic evaluation.

The pre-anesthetic evaluation revealed a patient classified as ASA 2, hemodynamically stable, with a normal preoperative workup. However, intraoperative respiratory management was deemed necessary, leading to the decision to use HFOT pre- and postoperatively. This approach facilitated preoxygenation with apneic ventilation and helped prevent acute postoperative respiratory failure.

**Figure 2:**
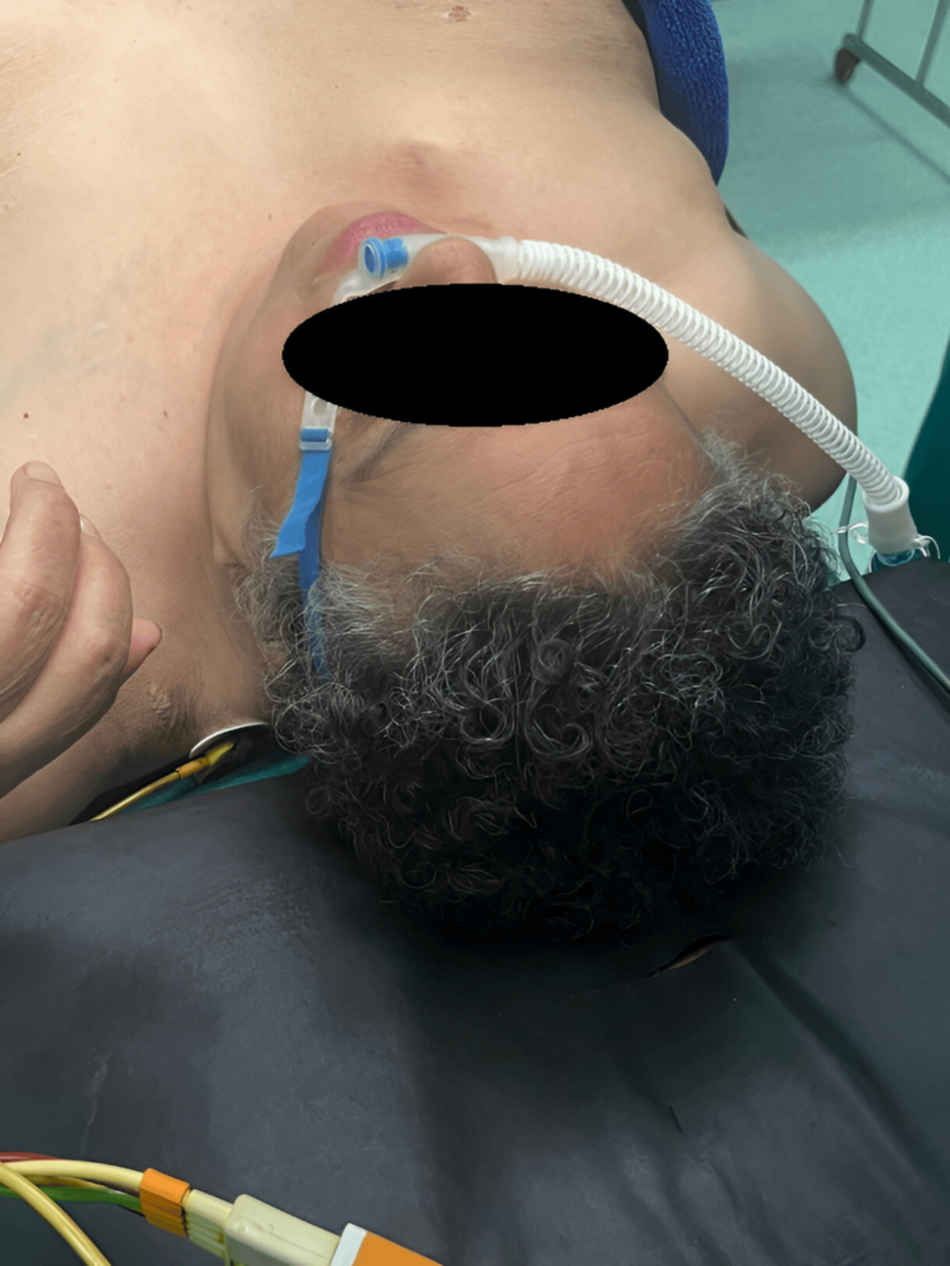
Preoperative preoxygenation with high-flow oxygen therapy (HFOT)

**Figure 3:**
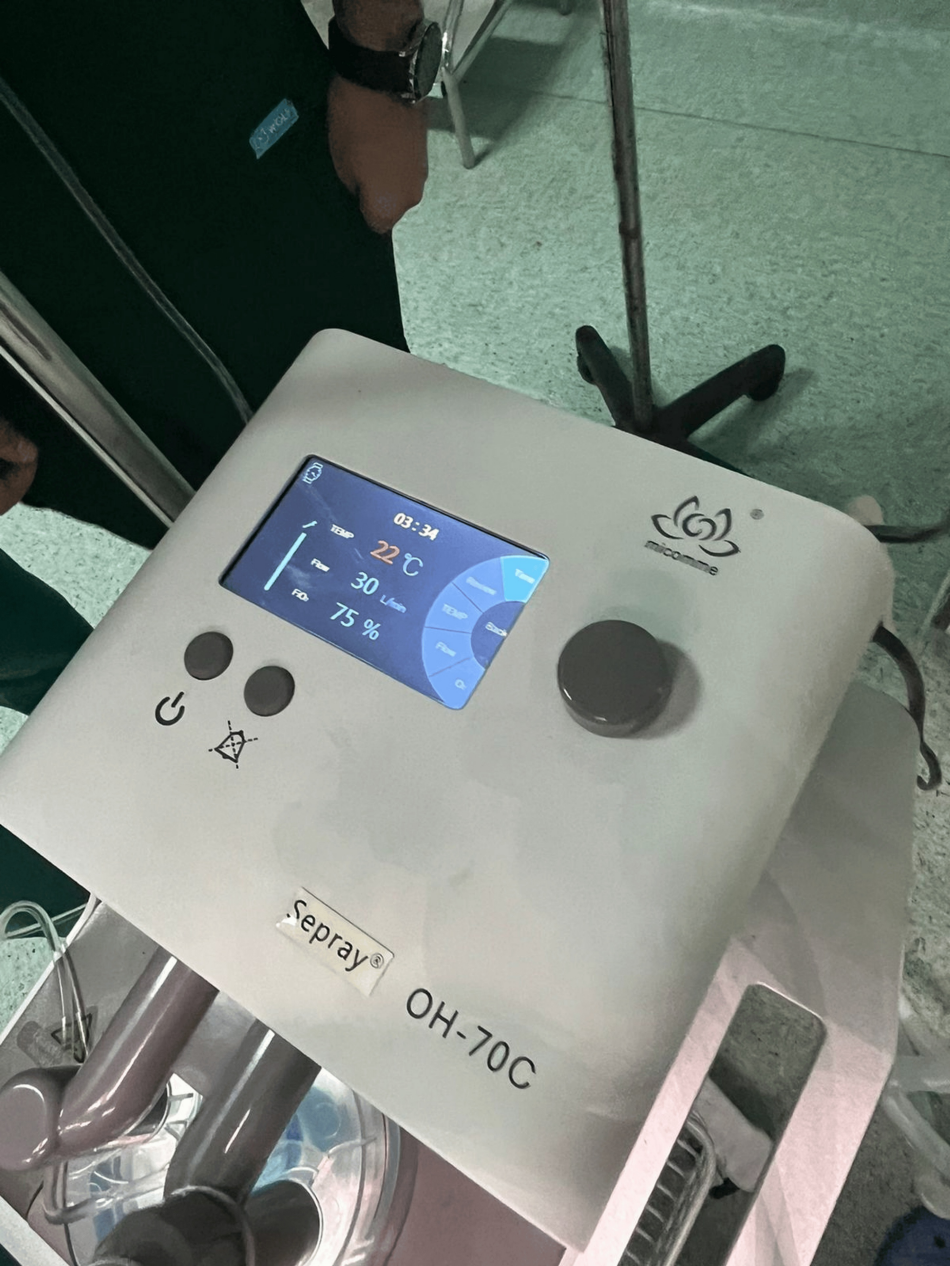
High-flow oxygen therapy (HFOT) parameters during preoxygenation

After her admission, the patient was positioned in a head-up (proclive) position, and monitoring, including heart rate (HR), blood pressure (BP), and oxygen saturation (SpO₂), was initiated. Vascular access was secured via a 16-G peripheral venous line, and vascular filling was performed with 500 mL of 0.9% saline solution. The subsequent steps of conditioning included the implementation of HFOT for preoxygenation (Figure 2), with a gradually increasing flow rate reaching up to 60 L/min (Figure 3).

**Figure 4:**
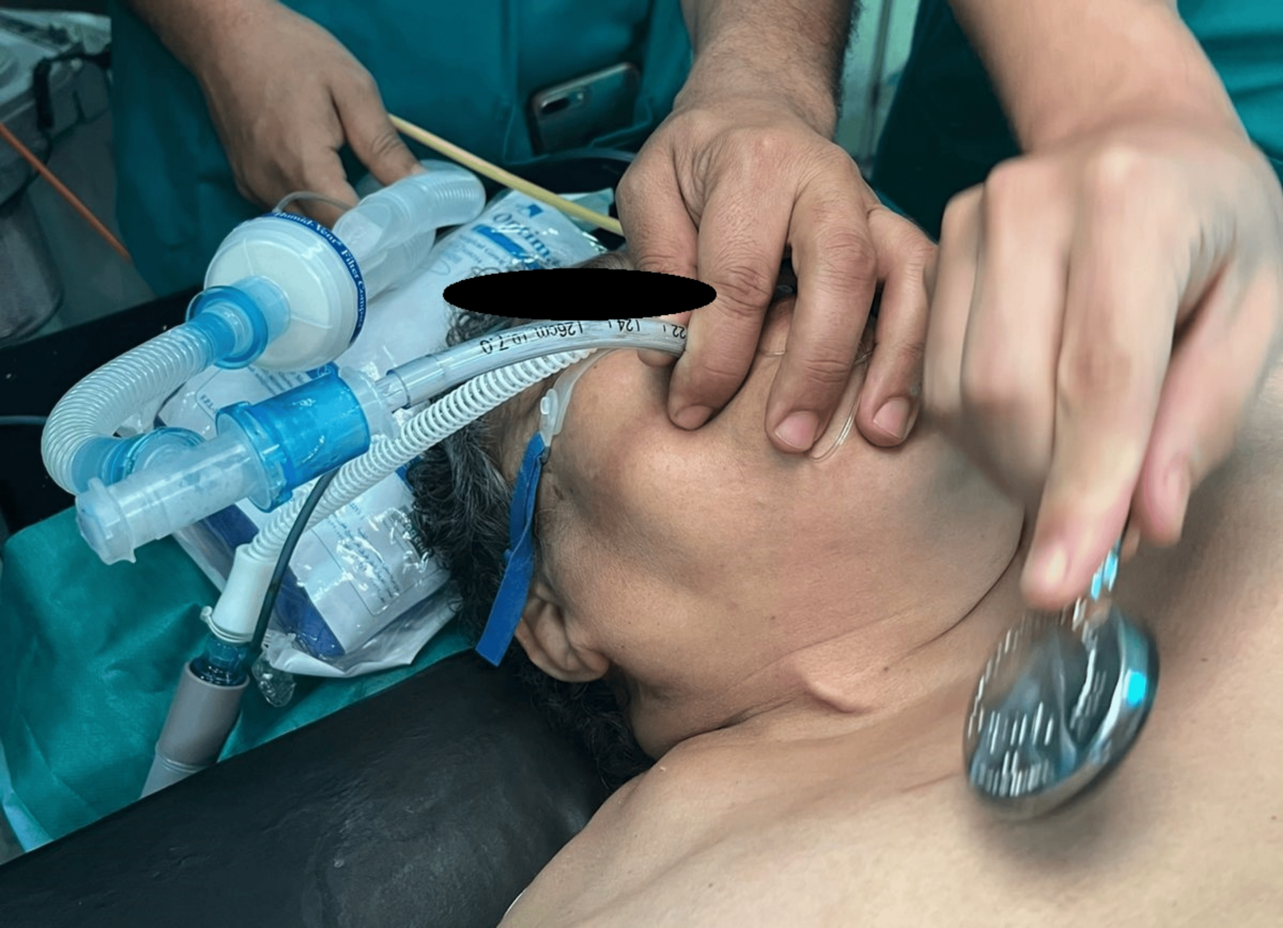
High-flow oxygen therapy (HFOT) during apneic ventilation and orotracheal intubation

**Figure 5:**
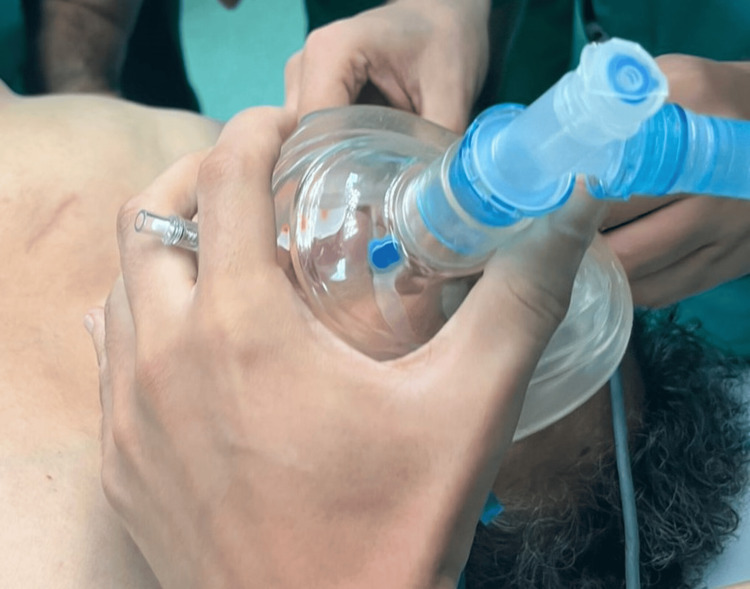
Combined preoxygenation using high-flow oxygen therapy (HFOT) and mask ventilation

After preoxygenation using both mask ventilation combined with HFOT (Figure 4), anesthetic induction was performed with 60 mg of lidocaine, 200 μg of fentanyl, 150 mg of propofol, and 50 mg of rocuronium; however, the desaturation was severe and brutal SpO_2_ before intubation remained to 60%, quickly followed by uncomplicated orotracheal intubation with a 7 mm endotracheal tube, while maintaining HFOT during apneic ventilation (Figure 5). The saturation remained stable at 98-100%.

**Figure 6:**
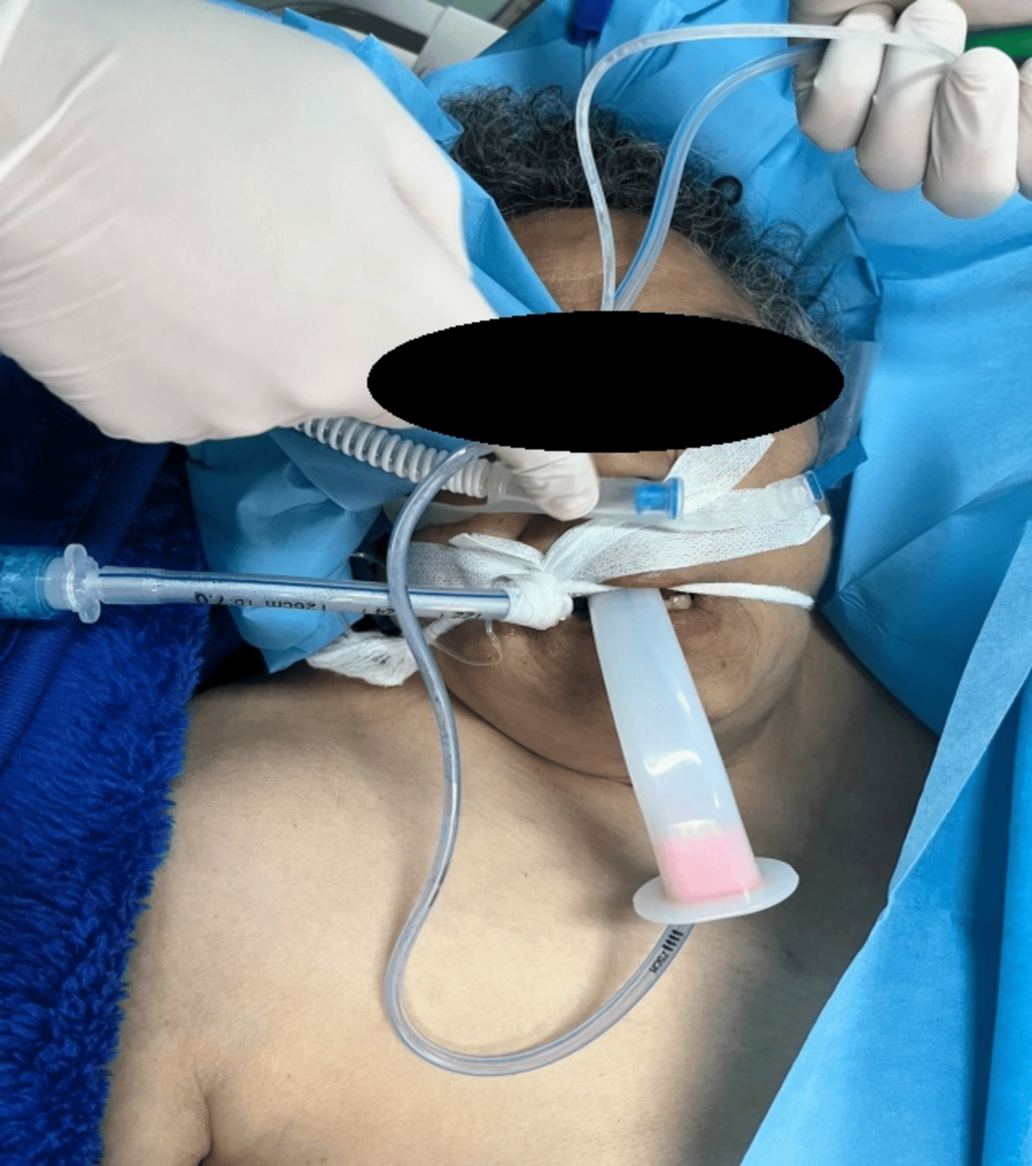
Extubation under high-flow oxygen therapy (HFOT)

The surgical procedure consisted of thoracoscopy with talc pleurodesis. During the 1.5-hour procedure, the saturation remained stable under protective mechanical ventilation with a tidal volume (Vt) of 6 mL/kg and FiO₂ at 50%. At the end of the surgery, the decision was made to implement HFOT to prevent postoperative respiratory distress, thereby facilitating the transition to spontaneous ventilation (Figure 6) with extubation under HFOT (Figurs 7).

**Figure 7:**
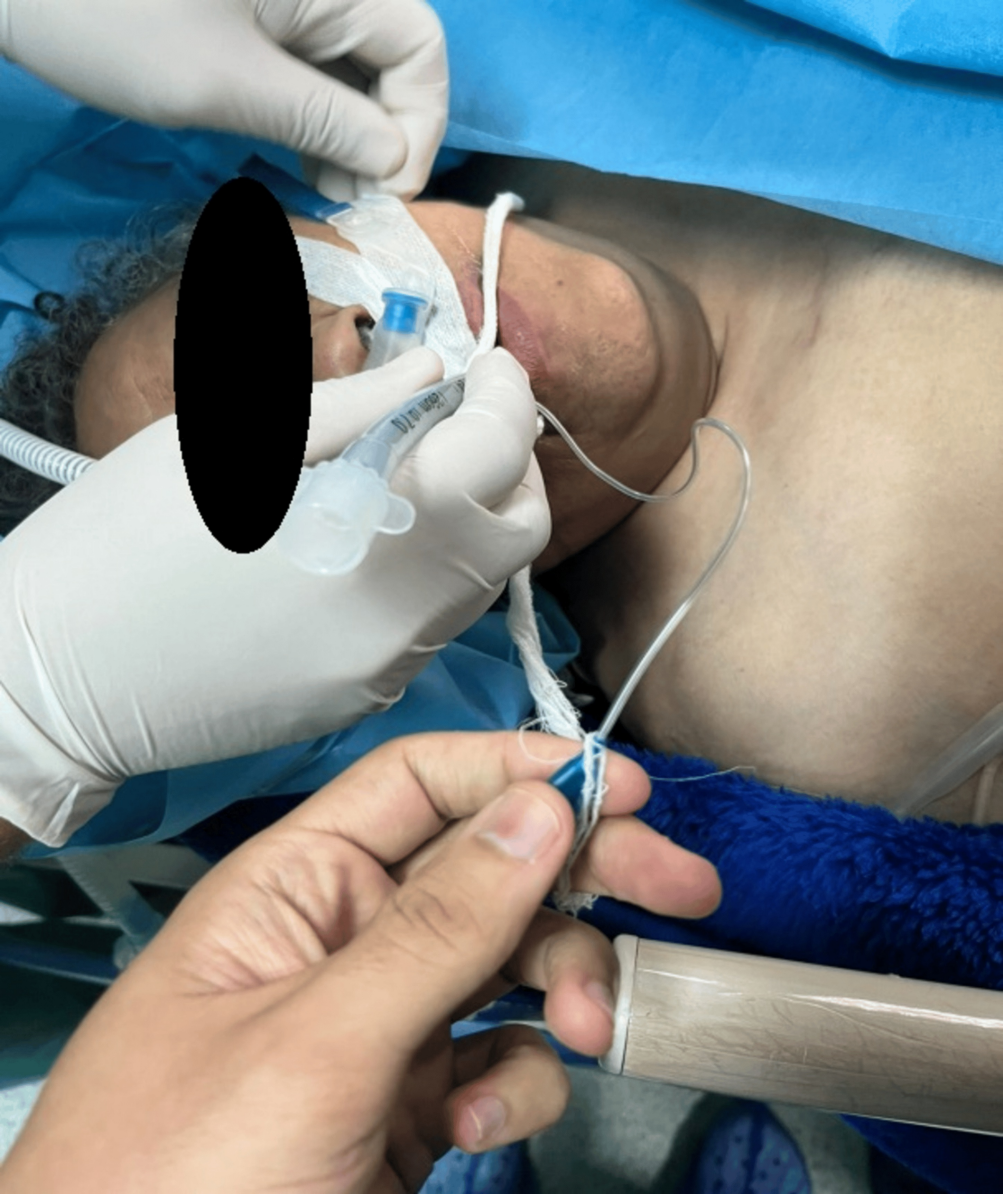
Extubation under high-flow oxygen therapy (HFOT) in spontaneous ventilation

Postoperatively, the patient’s saturation was normal at 98% two hours after HFOT, with a gradual reduction in the flow rate, eventually reaching 6 L/min. This allowed the patient to be transferred to the thoracic surgery department, avoiding a stay in the postoperative intensive care unit (ICU).

## Discussion

HFOT is a device that allows for the delivery of gas at high flow rates through the nasal route. This gas is heated and humidified, and its inspired oxygen fraction is adjustable. The physiological effects are numerous: washing of the anatomical dead space, stability of the inspired oxygen fraction, preservation of the mucosa, and maintenance of mucociliary clearance. These physiological effects explain the observed clinical benefits: improved gas exchange, reduced work of breathing, and enhanced comfort for the patient. Apneic oxygenation is a well-documented physiological phenomenon that allows for an increased duration of apnea without desaturation. This phenomenon explains the effectiveness of HFOT during anesthetic induction. Apneic oxygenation with HFOT has proven useful in patients at risk of difficult intubation, during rapid sequence induction, and also in pregnant women. In obese patients, combined preoxygenation with apneic oxygenation is less effective than non-invasive ventilation (NIV). The theoretical advantages of HFOT in the context of intubation in the operating room are twofold. On the one hand, we would benefit from the physiological advantages of HFOT described above, improving the quality of preoxygenation. On the other hand, HFOT allows for the continuation of apneic oxygenation during intubation. Indeed, the period between anesthetic induction (start of apnea) and the start of ventilation (end of apnea) is a critical period where potential complications of intubation, especially hypoxemia, can occur. By reducing the risk of profound hypoxemia, complications such as cardiac arrest could be avoided. The preoxygenation methods described above do not allow for continued oxygenation during intubation, as they require the use of a face mask that must be removed for laryngoscopy [8].

In the ICU, Miguel-Montanes et al [9]. compared three minutes of preoxygenation with a high-concentration mask and preoxygenation with HFOT at 60 L/min in patients with mild to moderate hypoxemia. HFOT achieved the lowest median SpO₂ during intubation at 100%, whereas it was 94% with the high-concentration mask. In an emergency department, two studies demonstrated the benefit of apneic oxygenation through nasal cannulas (at heterogeneous flow rates) during intubation [10,11]. Apneic oxygenation was associated with a higher incidence of successful intubation without desaturation. By contrast, two randomized trials did not show any difference between preoxygenation with HFOT or with a high-concentration mask in ICU patients [12,13]. The median lowest saturation value was statistically similar between the HFOT and mask preoxygenation groups. However, the HFOT flow rates varied in the studies (from 15 to 60 L/min). As discussed earlier, the physiological benefits of HFOT are demonstrated only at high flow rates (>40 L/min). In addition, the patients included were somewhat different, with varying degrees of hypoxemia.

In the operating room, many authors have investigated HFOT for preoxygenation and apneic oxygenation during intubation. This method of oxygenation is referred to as THRIVE in the literature ("transnasal humidified rapid insufflation ventilatory exchange"), particularly in its use for apneic oxygenation.

Since HFOT is a flow-based oxygenation technique, it cannot be used alone for preoxygenation. However, HFOT can be combined with an occlusive mask [14,15], especially in patients with hypoxemia. In such cases, it is delivered with a maximum FiO₂ of 100%. HFOT is well tolerated by awake patients, even at high flow rates (60 L/min) [16]. HFOT can be continued until the patient is intubated. It helps extend the time before desaturation during the apneic oxygenation phase, which corresponds to the period of intubation when the patient is in apnea. This feature is particularly useful during rapid sequence intubation, where the patient is not ventilated with a mask before intubation [17]. During the intubation phase, HFOT also facilitates CO₂ clearance, particularly during the first 20 minutes [17]

HFOT is increasingly used as an alternative to conventional oxygen therapy in the postoperative period, even before the onset of acute respiratory distress. In a study of 220 patients at moderate to high risk of postoperative pulmonary complications following major abdominal surgery, early preventive application of HFOT after extubation showed no difference in the incidence of hypoxemia compared to standard oxygen therapy. The authors concluded that HFOT was not superior to conventional oxygen therapy in non-hypoxemic patients after major abdominal surgery.

In the study by Stephan et al. [18], conducted in 830 patients postoperatively after thoracic surgery (either for prevention or treatment of acute respiratory failure), HFOT was not inferior to NIV for reducing a composite outcome that included the need for reintubation within 72 hours or a change in oxygen therapy method. The study by Hernandez et al. [19], which focused on ICU patients at high risk of extubation failure (including 40% of patients postoperatively), showed that preventive HFOT was not inferior to preventive NIV regarding the rates of reintubation and acute respiratory failure post-extubation.

In total, the 2020 European recommendations from the European Space Agency (ESA) and European Society of Intensive Care Medicine (ESICM) on non-invasive respiratory support for hypoxemic patients in the perioperative period recommend the use of HFOT in cases of intolerance to NIV or CPAP, in hypoxemic patients [20].

## Conclusions

Although HFOT has shown significant benefits in various areas of perioperative medicine, its use in thoracic surgery remains poorly defined due to a lack of specific data and robust clinical studies in this field. Thoracic surgery, due to its particularities such as the increased risk of respiratory complications, including hypoxemia and atelectasis, presents a major challenge in respiratory management. Despite growing evidence of the benefits of HFOT in the preoperative phase and for apneic oxygenation in other surgical specialties, studies examining its effectiveness and role in thoracic surgery are limited. Few trials have specifically investigated its use, and protocols vary depending on local practices, making it difficult to establish clear recommendations. As a result, although HFOT has the potential to improve oxygenation and reduce the risk of postoperative complications, further research is needed to better define its role in this specialty, particularly regarding optimizing post-surgical ventilation and preventing acute respiratory failure.
